# Evaluation of Sustainability Information Disclosure Based on Entropy

**DOI:** 10.3390/e20090689

**Published:** 2018-09-10

**Authors:** Ming Li, Jialin Wang, Ying Li, Yingcheng Xu

**Affiliations:** 1 School of Business Administration, China University of Petroleum-Beijing, Beijing 102249, China; 2 China National Institute of Standardization, Beijing 100191, China

**Keywords:** sustainability information disclosure, entropy, entropy weight, intuitionistic fuzzy sets

## Abstract

Disclosure of sustainability information is important for stockholders and governments. In order to evaluate the quality of sustainability information disclosure in heavily polluting industries, the quality of the disclosure is proposed to be evaluated from completeness, adequacy, relevance, reliability, normativeness and clarity aspects. The corresponding evaluation indicator system is constructed. Due to the ambiguity and complexity of the evaluation information, the intuitionistic fuzzy sets are applied to model the linguistic ratings. Entropy is used to derive the weight of experts, the object weight and the subject weight of the indicators. which are integrated when dealing with the evaluation information. The quality of sustainability information disclosure of seven representative companies in heavily polluting industries is evaluated. The importance of indicators and ranking of the companies are derived. Based on the evaluation results, the discussion and suggestions are also provided.

## 1. Introduction

In recent years, the sustainability has attracted a significant attention [1,2,3]. Sustainability information is used more and more in different stakeholders’ decisions [4]. Companies are advised to voluntarily disclose matters regarding their sustainability. The disclosure improves the accountability and transparency of companies’ operations and make the investors’ valuation proper [5]. More and more companies have begun to pay attention to their sustainability information disclosure [6,7,8,9,10,11]. For heavily polluting industries, its environmental impact, employees’ occupational health plans, and product safety issues are part of sustainability information. Therefore, the quality of sustainability information disclosure in heavily polluting industries are more concerned.

Since the disclosure of sustainability information is not compulsive, the quality of disclosed sustainability information is various [12,13,14]. High quality disclosure indicates that enterprises are willing to shoulder social responsibilities and establish a good corporate image. It will help attract investment, improve risk management ability, and enhance enterprise management performance [6]. The quality of the disclosure of sustainability information needs to be evaluated. Using the evaluation results, the stakeholder can comprehensively and directly understand the sustainability of the company. Meanwhile, it is also the valuable reference for the improvement of disclosure of sustainability information.

Researches have been put on the quality of sustainability information disclosure. Most focus on the impact of company or industry characteristics on the quality of sustainability information disclosure. For example, Brammer and Pavelin studied the impact of the nature of business activities, the environmental performance, firm size, company ownership etc. on the quality of corporate environmental information disclosure [6]; Orazalin and Mahmood used the largest oil and gas company in Russia as a sample to study the potential impacts of sustainability information quality [7]; Martínez-Ferrero et al., reveals the effect of financial reporting quality on sustainability information disclosure [9]. Michelon & Parbonetti examine the relationship of board composition, leadership and structure on sustainability disclosure [10]. Cuadrado-Ballesteros et al. study the relationship between the media pressure and the disclosure of sustainability information [11]. Dilling studies the characteristics of corporations that impact the quality of sustainability reports [15].

The research on the evaluation of quality of disclosures is relatively few. For example, Romolini et al., used inductive methods to measure the quality of disclosures in sustainability reports by assessing current disclosures of Global Reporting Initiative (GRI) indicators [13]; Manes-Rossi et al., selected 50 European companies to assess their disclosure levels by studying compliance with their annual reports and comprehensive reports on EUG [14]. The evaluations in these researches are based on the compliance of case companies to relevant indicators. It only evaluates from the integrity and standardization aspects, which are not complete. In fact, there are many other indicators to be paid attention to. For example, the accordance with relevant indicators can only reflects whether corresponding contents are disclosed. The degree of detail of the disclosure cannot be reflected. Obviously, different level of detail of the disclosure will lead to significant differences in the quality of the disclosure of sustainability information. But the in previous methods, it cannot be reflected. Moreover, the evaluation is performed by one or two persons [13,14]. As very few people participated in the evaluation, there is probably bias in the judgement due to limited levels of expertise. Aggregating the opinions of multiple experts will ease the bias of a single person to the greatest extent and make the evaluation more objective.

In order to resolve the problem, this paper proposed the approach to evaluating the quality of sustainability information disclosure. With the proposed method, the quality of the disclosure can be evaluated directly and more comprehensively.

Firstly, aspects of the evaluation are determined, which guides the construction of the indicator system. It focuses more on the quality of information disclosure. Then the corresponding indicators tied to sustainability for the evaluation of quality of sustainability information disclosure is derived. It is fully considered that the contents of the evaluation are sustainability information. With the analysis and extension of the existing literatures [8,16,17,18], the derived six aspects are completeness [16], adequacy [8], relevance [17], reliability [17], normativeness [18] and clarity [17]. Completeness refers to the breadth of the report disclosure [16]. Adequacy reflects the depth of disclosure of the report. Relevance shows the usefulness of the report disclosure to the reader [8]. Reliability is the trustworthiness of the reader to the content of the report [17]. Normativeness describes the compliance of the report with the G3.1 indicator disclosure requirement [18]. Clarity indicates the reader’s ability to comprehend the sustainability reporting [17].

Afterwards, the novel evaluation method is proposed to deal with the evaluation information, which not only considers the weight of experts but also subject and object weights of indicators. In the method, a group of experts are invited to give the evaluation information. Since the linguistic terms are preferred in the evaluation, the intuitionistic fuzzy sets are used to model the linguistic ratings. Not only the membership and nonmembership but also the hesitation is used to characterize the vagueness and uncertainty [19,20,21,22]. The evaluation information can be modeled more comprehensively. Different expertise and knowledge lead to different level on the accuracy of opinions. The weights of experts are calculated based on entropy [23]. Moreover, indicators play different roles in the discrimination of candidates. The subject weight representing the importance and the object weight representing the discrimination capability are often integrated as the weight of indicators [24,25]. However, in intuitionistic fuzzy setting, they are treated separately in current researches [23,26]. In the study, the two kinds of weights are integrated based on entropy. Finally, the opinions of multiple experts are integrated as the final evaluation results with the weight of experts and indicators.

Finally, the seven representative companies are selected as the case company to verify the proposed approach. Meanwhile, the evaluation results are analyzed and suggestions are given for further improvement.

The rest of this paper is organized as follows. Section 2 introduces the entropy and intuitionistic fuzzy sets. Section 3 builds evaluation indicator system from six aspects. Section 4 proposes the evaluation method. Section 5 is the application of the proposed approach. The conclusion along with future research are given in Section 6.

## 2. Entropy and Intuitionistic Fuzzy Sets

In decision making, exact numeric values are always difficult obtained because of the uncertainty and fuzziness [27,28,29,30,31,32]. In order to resolve the problem, the intuitionistic fuzzy set was newly put forward [33]. Intuitionistic fuzzy set is the generalization of fuzzy set. In fuzzy set, the membership of an element to a fuzzy set is ranging from zero to one [34,35,36,37]. But sometimes, the sum of the degree of membership and the degree of nonmembership may not be equal to 1. There may be some hesitation. Therefore, the degree of non-membership is added to the fuzzy set to construct intuitionistic fuzzy set [33]. It is more useful in dealing with vagueness and uncertainty and has been widely used in decision situations [22,26,38,39]. Considering the ratings in the study are in the linguistic form, the intuitionistic fuzzy set is applied to model the linguistic ratings more comprehensively. In order to discriminate the experts and indicator, the entropy, which is proposed to measure the discriminatory power in decision making [40,41,42,43,44,45], is used. Entropy and intuitionistic fuzzy sets are reviewed briefly as follows [40,41,46,47,48,49,50].

**Definition** **1.** *[49]. Let X be a given finite set, an intuitionistic fuzzy set on X can be defined as A.*

$$A=\{\left[x,\;{µ}_{A}\left(x\right),\;v_{A}\left(x\right)\right]\;|x\in X$$

*where $0\leq {µ}_{A}\left(x\right)\leq 1$ and $0\leq v_{A}\left(x\right)\leq 1$ respectively represents the membership function and the non-membership function, and $0\leq {µ}_{A}\left(x\right)+v_{A}\left(x\right)\leq 1$. While $\pi_{A}\left(x\right)=1-{µ}_{A}\left(x\right)-v_{A}\left(x\right)$ represents the hesitancy degree of whether x belongs to A or not, where $0\leq \pi_{A}\left(x\right)\leq 1$.*

**Definition** **2.** *[50]. Intuitionistic fuzzy number operators.*

*Assume $\alpha =\left({µ}_{A},v_{A}\right)\;$ and $\beta =\left({µ}_{B},v_{B}\right)$ be the intuitionistic fuzzy numbers, and λ be the real number. Then the intuitionistic fuzzy number algorithms on α and β are defined as follows.*

(1) $$\alpha ⊕\beta =\left({µ}_{A}+{µ}_{B}-{µ}_{A}{µ}_{B},\;\;v_{A}v_{B}\right),$$

(2) $$\alpha ⊗\beta =\left({µ}_{A}{µ}_{B},\;\;v_{A}+v_{B}-v_{A}v_{B}\right),$$

(3) $$\lambda \alpha =\left(1-{\left(1-{µ}_{A}\right)}^{\lambda },\;\;v_{A}{}^{\lambda }\right),\;\lambda >0,$$

(4) $$\alpha^{\lambda }=\left({µ}_{A}^{\lambda },\;1-{\left(1-v_{A}\right)}^{\lambda }\right),\;\lambda >0,$$

(5) $$\frac{\alpha }{\beta }=\left(min({µ}_{A},{µ}_{B}\right),\;\max\left(v_{A},v_{B}\right)),$$

**Definition** **3.** *[40] Intuitionistic fuzzy entropy.*

*For intuitionistic fuzzy set $A=\{\left[x_{i},\mu_{A}\left(x_{i}\right),v_{A}\left(x_{i}\right)\right]\;|x_{i}\in U\}\in IFS\left(U\right)$, $U=\left\{x_{1},x_{2},\ldots ,x_{m}\right\}$, the intuitionistic fuzzy entropy of A is defined as follows.*

(6) $$E\left(A\right)=\frac{1}{n}\sum_{i=1}^{n}\left[1-\sqrt{{\left(1-\pi_{A}\left(x_{i}\right)\right)}^{2}-\mu_{A}\left(x_{i}\right)v_{A}\left(x_{i}\right)}\right],$$

*where $\pi_{A}\left(x_{i}\right)=1-{µ}_{A}\left(x_{i}\right)-v_{A}\left(x_{i}\right)$, $\forall x_{i}\in U$.*

**Definition** **4.** *[41] Entropy weight.*

*The entropy weight of jth indicator is defined as follows.*

(7) $$w_{j}=\frac{1-E\left(A_{j}\right)}{\sum_{j=1}^{n}\left(1-E\left(A_{j}\right)\right)},$$

*where $E\left(A_{j}\right)$ is the intuitionistic fuzzy entropy of jth indicator.*

**Definition** **5.** *[40] For intuitionistic fuzzy number, the score function of α is defined as follows.*

(8) $$s\left(\alpha \right)=k{µ}_{\alpha }-kv_{\alpha }+\left(1-2k\right)\left|1-\left(µ+v_{\alpha }\right)\right|,$$

*where k reflects the decision attitude of the evaluator, and 0<k<1. When 1/2<k<1, the evaluator is optimistic; when 0<k<1/2, the evaluator is pessimistic; and k=1/2 respects the evaluator is neutral.*

## 3. Construction of Indicator System

Sustainability information disclosure essentially belongs information disclosure related to sustainability. Therefore, there needs to determine the aspects of information disclosure firstly. Based on the literatures [8,16,17,18], the six aspects including completeness [16], adequacy [8], relevance [17], reliability [17], normativeness [18] and clarity [17] are determined. In the work [16], the completeness is used to measure the environmental information disclosure. The adequacy is used in the work [8] to evaluate the environmental performance information disclosure. Relevance, reliability and clarity are used for the evaluation of quality of social responsibility information disclosure [17]. Normativeness is derived from the work [18], which is used to assess the quality of sustainability reporting.

Based on the above aspects and the information tied to sustainability, the sustainability information disclosure quality evaluation indicator system is built, which is directly related to sustainability information disclosure. For example, sustainability information usually includes social, economic and environmental aspects. Therefore, in the aspect of completeness, there are the degree of disclosure of economic information and social information. The environmental information is subdivided into dye emissions, total emission reduction. It can be seen that the indicators are closely related to sustainability information.

The quality evaluation indicator system for sustainability information disclosure is shown in Table 1 and the detailed illustration are as follows.

(1) Completeness [16]

Completeness refers to the breadth of the report disclosure. Sustainability reports generally cover social, economic and environmental aspects [12]. For the heavily polluting industry, the most important is its environmental information. In the “Guidelines for Environmental Information Disclosure of Listed Companies” (Draft for Comment) issued by the Ministry of Environmental Protection of China in 2010 [51], the 16 heavily polluting industries identified by their classification must disclose the degree of disclosure of pollutant discharge compliance, the degree of disclosure of the completion of the total emission reduction task, the degree of disclosure of the implementation of the “three simultaneous” system etc [16].

(2) Adequacy [8]

Adequacy refers to the depth of disclosure of the report. For heavily polluting enterprises, the part that should be substantially disclosed is the environmental part. Generally speaking, the larger the proportion of environmental information, the more adequate the information disclosure. In addition, the way information is disclosed also determines the adequacy of information disclosure to a certain extent. At present, there are three main ways of environmental information disclosure, including consolidated reports, supplementary reports and independent reports [16]. Compared with consolidated reports and supplementary reports, corporate disclosures using independent reports will be more fully disclosed.

(3) Relevance [17]

Relevance is the usefulness of the report disclosure to the reader, which generally includes three aspects: timeliness [17], predictability and importance [52]. For matters happening in the enterprise, timely disclosure should be made. The more timely the disclosure, the higher the quality of the report [17]. The content of the report should be predictive. For heavily polluting industries, the predictability is mainly reflected in whether the expected environmental risks are disclosed. In addition, issues of concern to stakeholders should be substantively disclosed in accordance with the principle of importance.

(4) Reliability [17,53]

Reliability is the trustworthiness of the reader to the content of the report. Mainly depends on the neutrality, verifiability and authenticity of the report [17]. In addition, whether the company passes the ISO (International Organization for Standardization) environmental system certification [54] and reports whether it is audited by an independent third party will also affect the reliability of the report. For heavily polluting enterprises, environmental problems are the most important problems they face, and the ISO environmental system certification can prove that the organization has reached an international level in environmental management, which can enhance readers’ conviction on reports. And if the report is audited by an independent third party, it can also enhance the reliability of the report and improve the quality of the report.

(5) Normativeness [8,18]

Currently, there are no uniform disclosure standards for sustainability reports. Different companies have different disclosure standards, guidelines, and specifications. In addition, the reporting language also has problems such as normativeness and rigor. In recent years, as GRI guidelines have become more widely used, more and more companies are providing GRI indicator index in their report appendix [8]. This index describes the compliance of the report with the G3.1 indicator disclosure requirements, which to some extent enhances the normative nature of the report and improves the quality of the report.

(6) Clarity [17]

Clarity is the reader’s comprehensibility of sustainability reporting. When preparing a sustainability report, it is necessary to give practical consideration to the reader’s ability to understand [17,54]. If the disclosed report is difficult to understand, it loses its meaning. Therefore, it is necessary to use an easy-to-understand language when disclosing, and to make necessary explanations for unavoidable technical terms and abbreviations. In addition, the sustainability report should not be limited to a fixed form. Personalized disclosure should be encouraged to avoid the same content that is disclosed each year, so that readers can have a deeper understanding of the sustainable development of the company through reading reports [17].

## 4. Evaluation Method

The indicators in Table 1 are qualitative. It is difficult to get exact numerical judgements. Instead, linguistic assessments are preferred. Because the ratings are in linguistic form, intuitionistic fuzzy sets are applied to model the linguistic ratings comprehensively. The intuitionistic fuzzy entropy is used to discriminate the experts and indicators. Since expertise level and familiarity degree are probably not identical, experts need to be discriminated [23]. The stronger consistency of opinions indicates the expert is more reliable [23]. The corresponding expert will be given a higher weight. The two kinds of expert weights are derived by the ratings of enterprise and indicators respectively based on intuitionistic fuzzy entropy [23]. Moreover, the weights of indicators are also not same [24]. The weight of indicators should include the importance and the discrimination [24]. The importance named as subject weight is often rated by experts. The discrimination capability named as object weight is often derived by calculating the discrimination capability of the indicator. In intuitionistic fuzzy settings, these two kinds of weight are used separately in current researches [23,26]. In order to get a more comprehensive weight of indicators, in the study, the object weight [41] and subject weight [23,56] are derived with intuitionistic fuzzy entropy and integrated as the weight of indicators. Firstly, the weight of experts and the corresponding weighted score and weighted importance of the indicator are derived [23]. The object weight of the indicator is calculated [41]. Then the weighted importance and object weight of the indicator are integrated as the weights of the indicator [25]. Finally, the evaluation results are derived by aggregating ratings. The detailed steps are given as follows.

Let $E=\left\{e_{1},e_{2},\;\ldots ,e_{k}\right\}$ be the set of evaluators, $P=\left\{P_{1},P_{2},\;\ldots ,P_{i},\;\ldots ,P_{m}\right\}$ (i=1,2,…,m) be the set of alternatives, and $C=C_{1}\cup^{}C_{2}\cup^{}\cdots \cup^{}C_{l}=\left\{c_{1},c_{2},\;\ldots ,c_{j},\;\ldots c_{n}\right\}$ (j=1,2,…,n) be the set of indicators. Assume that the weight vector for the score with respect to indicator $c_{j}$ of m alternatives $P_{i}$ can be expressed in $\lambda ={\left(\lambda_{1},\lambda_{2},\ldots ,\lambda_{k}\right)}^{T}$, where $0\leq \lambda_{k}\leq 1,\;\;k=1,2,\ldots ,K$ and $\sum_{k=1}^{K}\lambda_{k}=1$, and the score for importance of indicator $c_{j}$ of enterprise $m_{s}$ can be expressed in $\delta ={\left(\delta_{1},\delta_{2},\ldots ,\delta_{k}\right)}^{T}$, where $0\leq \delta_{k}\leq 1,$ $\sum_{k=1}^{K}\delta_{k}=1$. The *K* evaluators are invited to evaluate the score with respect to indicator $c_{j}$ of enterprise $m_{s}$ and the score for importance of indicator $c_{j}$ of enterprise $m_{s}$ separately. Then obtain the intuitionistic fuzzy decision matrix $R^{k}={\left(r_{ij}^{k}\right)}_{m\times n}$ and $W^{k}={\left(w_{j}^{k}\right)}_{1\times n}$ by evaluator $e_{k}$, where the intuitionistic fuzzy set can be expressed in IFS (intuitionistic fuzzy sets) $\alpha_{ij}^{k}=\left(\mu_{ij}^{k},v_{ij}^{k}\right)$. And $\mu_{ij}^{k}$, $v_{ij}^{k}$, $\pi_{ij}^{k}$ respectively express the satisfaction, dissatisfaction and hesitancy degree of evaluator under the indicators $c_{j}$ of the enterprise $m_{s}$, $0\leq \mu_{ij}^{k}\leq 1$, $0\leq v_{ij}^{k}\leq 1$, $0\leq \mu_{ij}^{k}+v_{ij}^{k}\leq 1$, $\pi_{ij}^{k}=1-\mu_{ij}^{k}-v_{ij}^{k}$. 

**Step 1.** Assume that the scoring with respect to indicator $c_{j}$ of enterprise $P_{i}$ given by the evaluator $e_{k}$ can be expressed in IFS $r_{ij}^{\left(k\right)}=\left({µ}_{ij}^{\left(k\right)},v_{ij}^{\left(k\right)},\pi_{ij}^{\left(k\right)}\right)$, the scoring for importance of indicator $c_{j}$ given by the evaluator $e_{k}$ can be expressed in IFS $w_{j}^{\left(k\right)}=\left({µ}_{j}^{\left(k\right)},v_{j}^{\left(k\right)},\pi_{j}^{\left(k\right)}\right)$. Therefore, the corresponding intuitionistic fuzzy decision matrix $R^{\left(k\right)}$ and $W^{\left(k\right)}$ can be shown concisely in matrix in the following form.

$$R^{\left(k\right)}\;=\;{\left(r_{ij}^{\left(k\right)}\right)}_{m\times n}\;=\;\begin{array}{cc} & \;\begin{array}{c} \begin{array}{cccccc} c_{1}\; & c_{2}\; & \ldots & c_{j\;} & \ldots & \;c_{n} \end{array} \end{array}\;\\ \;\begin{array}{c} P_{1}\\ \begin{array}{c} P_{2}\\ \begin{array}{c} \vdots \\ P_{i}\\ \vdots \\ P_{m\;} \end{array} \end{array} \end{array}\; & \begin{array}{cc} & \left[\begin{array}{cccccc} r_{11}^{\left(k\right)} & r_{12}^{\left(k\right)} & \ldots & r_{1j}^{\left(k\right)} & \ldots & r_{1n}^{\left(k\right)}\\ r_{21}^{\left(k\right)} & r_{22}^{\left(k\right)} & \ldots & r_{2j}^{\left(k\right)} & \ldots & r_{2n}^{\left(k\right)}\\ \vdots & \vdots & \vdots & \vdots & \vdots & \vdots \\ r_{i1}^{\left(k\right)} & & & r_{ij}^{\left(k\right)} & \ldots & r_{in}^{\left(k\right)}\\ \vdots & & \vdots & \vdots & & \vdots \\ r_{m1}^{\left(k\right)} & r_{m2}^{\left(k\right)} & \ldots & r_{mj}^{\left(k\right)} & \ldots & r_{mn}^{\left(k\right)} \end{array}\right] \end{array} \end{array}$$

$$\;W^{\left(k\right)}=\left(w_{1}^{\left(k\right)},w_{2}^{\left(k\right)},\ldots ,w_{j}^{\left(k\right)},\ldots ,w_{n}^{\left(k\right)}\right)\;$$

where $r_{ij}^{\left(k\right)}=\left({µ}_{ij}^{\left(k\right)},v_{ij}^{\left(k\right)},\pi_{ij}^{\left(k\right)}\right)$, $w_{j}^{\left(k\right)}=\left({µ}_{j}^{\left(k\right)},v_{j}^{\left(k\right)},\pi_{j}^{\left(k\right)}\right)$.

**Step 2.** Obtain intuitionistic fuzzy entropy of the scoring of enterprise. According to the evaluator evaluation results of the scoring with respect to indicator $c_{j}$ of alternative $P_{i}$, calculate the intuitionistic fuzzy entropy $E_{k}^{P_{i}}$ (k=1,2,…,K,  i=1,2,…,m) and then obtain the entropy weight of n indicators [56] as the weight of evaluator $\lambda_{k}^{P_{i}}$ on the enterprise score by the following formula.

(9) $$E_{k}^{P_{i}}=\frac{1}{n}\sum_{j=1}^{n}\left[1-\sqrt{{\left(1-\pi_{ij}^{\left(k\right)}\right)}^{2}-{µ}_{ij}^{\left(k\right)}v_{ij}^{\left(k\right)}}\right],$$

(10) $$\lambda_{k}^{P_{i}}=1-E_{k}^{P_{i}}/\left(K-\sum_{k=1}^{K}E_{k}^{P_{i}}\right),$$

**Step 3.** Calculate the weighted evaluation value for the scoring of enterprise. Based on the weight of kth evaluator on the enterprise score $\lambda_{k}$ and the enterprise scoring information, with Equation (3), calculate the weighted evaluation value of indicators $c_{j}$ of enterprise $P_{i}$ on the enterprise scoring $\eta_{j}^{P_{i}}$.

(11) $$\eta_{j}^{P_{i}}=\sum_{k=1}^{K}r_{ij}^{\left(k\right)}\lambda_{k}=\left(\rho_{kj},\sigma_{kj}\right),$$

where $\rho_{ij}=1-\prod_{k=1}^{K}(1-{µ}_{ij}^{\left(k\right)}){}^{\lambda_{k}}\;,\;\sigma_{kj}=\prod_{k=1}^{K}(v_{ij}^{\left(k\right)}){}^{\lambda_{k}}$.

**Step 4.** Obtain the entropy weight on the enterprise scoring. Based on the weighted evaluation value $\eta_{j}^{P_{i}}=\left(\rho_{kj},\sigma_{kj}\right)$, with Equations (6) and (7), calculate the entropy weight $\gamma_{j}$ with respect to indicators $c_{j}$ of all m enterprises on the enterprise scoring by using the following formula [41].

(12) $$E_{j}^{P_{i}}=\frac{1}{m}\sum_{i=1}^{m}\left[1-\sqrt{(1-{\left(1-\rho_{kj}-\sigma_{kj}\right)}^{2}-\rho_{kj}\sigma_{kj}}\right],$$

(13) $$\gamma_{j}=\frac{1-E_{j}^{P_{i}}}{\sum_{j=1}^{n}\left(1-E_{j}^{P_{i}}\right)},$$

**Step 5.** Obtain intuitionistic fuzzy entropy of the scoring for importance of indicators. According to the evaluator evaluation results of the scoring for importance of indicator $c_{j}$ of enterprise $P_{i}$, calculate the intuitionistic fuzzy entropy $E_{k}$ (k=1,2,…,K) and then obtain the entropy weight of n indicators [56] as the weight of evaluator $\delta_{k}$ on the scoring for importance of indicator by the following formula.

(14) $$E_{k}=\frac{1}{n}\sum_{j=1}^{n}\left[1-\sqrt{{\left(1-\pi_{j}^{\left(k\right)}\right)}^{2}-{µ}_{j}^{\left(k\right)}v_{j}^{\left(k\right)}}\right],$$

(15) $$\delta_{k}=1-E_{k}/\left(K-\sum_{k=1}^{K}E_{k}\right),$$

**Step 6.** Calculate the weighted evaluation value for the scoring for importance of indicators. Based on the weight of kth evaluator on the enterprise score $\delta_{k}$ and the importance of indicator scoring of enterprise $P_{i}$ information, with Equation (3), calculate the weighted evaluation value $\eta_{j}$ of indicators $c_{j}$ on the scoring for importance of indicator as the weight of indicators.

(16) $$\eta_{j}=\sum_{k=1}^{K}w_{j}^{\left(k\right)}\delta_{k}=\left(\rho_{j},\sigma_{j}\right),$$

where $\rho_{j}=1-\prod_{k=1}^{K}(1-{µ}_{j}^{\left(k\right)}){}^{\lambda_{k}}\;,\;\sigma_{j}=\prod_{k=1}^{K}(v_{j}^{\left(k\right)}){}^{\lambda_{k}}$.

**Step 7.** Obtain the weight of indicators. Based on the entropy weight of the enterprise scoring $\gamma_{j}$ and the weighted evaluation value $\eta_{j}$ for the scoring for importance of indicators, with Equation (3), obtain the weight of indicators $\beta_{j}$.

(17) $$\beta_{j}=\gamma_{j}\eta_{j}=\left(\rho_{j}^{\prime },\sigma_{j}^{\prime }\right),$$

**Step 8.** Calculate the weight of indicators. According to the weight of indicators $\beta_{j}$, with Equation (8), calculate the corresponding score value.

(18) $$s\left(\beta_{j}\right)=k\rho_{j}^{\prime }-k\sigma_{j}^{\prime }+\left(1-2k\right)\left|1-\left(\rho_{j}^{\prime }+\sigma_{j}^{\prime }\right)\right|,$$

**Step 9**. Obtain the final evaluation results. Based on the weight of enterprise scoring $\eta_{j}^{P_{i}}$ and the weight of indicators $\beta_{j}$, with Equation (2), obtain the final evaluation results by using the following formula.

(19) $$\xi_{i}^{P_{i}}=\sum_{c_{j}\in c_{n}}⊗\eta_{j}^{P_{i}}\;\beta_{j}=\sum_{c_{j}\in C_{l}}⊗\eta_{j}^{P_{i}}\;\beta_{j}={\left(\varepsilon_{i}\right)}_{m\times 1}=\left(\rho_{i},\sigma_{i}\right),$$

**Step 10.** Calculate the final evaluation score of m enterprise $P_{i}$. According to the final evaluation results $\xi_{i}^{P_{i}}$, with Equation (8), calculate the final evaluation score $s_{i}$ (i=1,2,…,m) of enterprise $P_{i}$ by using the score function as follows.

(20) $$s_{i}=k\rho_{i}-k\sigma_{i}+\left(1-2k\right)\left|1-\left(\rho_{i}+\sigma_{i}\right)\right|,$$

## 5. Illustrative Example

China is a developing country. Many of the resources used in heavily polluting industries are scarce and non-renewable, and many companies do not make efficient use of resources. Moreover, a large amount of pollution will be generated in the production process and cause great damage to the environment. Therefore, it is not doing well in terms of sustainable development. In recent years, as China’s attention to sustainable development has increased, the sustainability requirements for heavy polluting industries have increased accordingly. For some companies with serious pollution, they have also been rectified accordingly. Therefore, stakeholders are very concerned about sustainability information disclosure in heavily polluting industries. Therefore, from the heavy pollution industry, this paper selected seven representative companies for evaluation.

### 5.1. Evaluation

According to the “Guidelines for Environmental Information Disclosure of Listed Companies” (Draft for Comment) issued by the China’s Ministry of Environmental Protection in 2010 [51], heavy pollution industry is divided into 16 sub-sectors. We select seven industries that involve non-renewable energy as raw materials, and considers that these industries will pay more attention to their sustainability information, and select one representative enterprise from each of the seven sub-sectors to conduct research for the sample company. The enterprises to be evaluated in this article include Zijin Mining Group, Shandong Hongqiao, Baoshan Iron & Steel Co., Ltd., Shaanxi Coal and Chemical Industry Group, Sinopec Group, Conch Cement Co. Ltd., and Boway Alloy Material Co., Ltd. ($P_{1}$,$\;P_{2}$,$\;P_{3}$,$\;P_{4}$,$\;P_{5}$,$\;P_{6}$,$\;P_{7}$). A group of 12 experts ($e_{1}$,$\;e_{2}$,…,$\;e_{12}$) are invited to evaluate the importance of the indicators and score the seven enterprises according to indicators using the linguistic terms in Table 2. The following is the specific evaluation process.

**Step 1.** Construct the intuitionistic fuzzy decision matrix of information quality level of the enterprise and the intuitionistic fuzzy decision matrix of weight of indicators by using the linguistic terms in Table 2, based on the evaluation result of experts. The intuitionistic fuzzy decision matrices of weight of indicators obtained is shown in Table 3.

**Step 2.** Obtain the weight of experts on the enterprise score with Equations (9) and (10), where m=7, n=27, K=12. The result is shown in Table 4.

**Step 3.** Calculate the weighted evaluation value for the scoring of enterprise with Equations (11). The result obtained is shown in Table 5.

**Step 4.** Obtain the entropy weight on the enterprise scoring with Equations (12) and (13).

*γ*_1_ = 0.038, *γ*_2_ = 0.036, *γ*_3_ = 0.037, *γ*_4_ = 0.038, *γ*_5_ = 0.038, *γ*_6_ = 0.038, *γ*_7_ = 0.037, *γ*_8_ = 0.036, *γ*_9_ = 0.036, *γ*_10_ = 0.037, *γ*_11_ = 0.038, *γ*_12_ = 0.036, *γ*_13_ = 0.035, *γ*_14_ = 0.035, *γ*_15_ = 0.038, *γ*_16_ = 0.035, *γ*_17_ = 0.037, *γ*_18_ = 0.036, *γ*_19_ = 0.038, *γ*_20_ = 0.037, *γ*_21_ = 0.037, *γ*_22_ = 0.038, *γ*_23_ = 0.037, *γ*_24_ = 0.038, *γ*_25_ = 0.038, *γ*_26_ = 0.039, *γ*_27_ = 0.037

**Step 5.** Obtain intuitionistic fuzzy entropy of the scoring for importance of indicators with Equations (14) and (15).

$$E_{1}=0.137,E_{2}=0.200,E_{3}=0.137,E_{4}=0.137,E_{5}=0.166,E_{6}=0.104,E_{7}=0.134,\;E_{8}=0.101,E_{9}=0.110,E_{10}=0.118,E_{11}=0.125,E_{12}=0.129.$$

$$\delta_{1}=0.083,\delta_{2}=0.077,\delta_{3}=0.083,\delta_{4}=0.083,\delta_{5}=0.080,\delta_{6}=0.086,\delta_{7}=0.083,\;\delta_{8}=0.086,\delta_{9}=0.086,\delta_{10}=0.085,\delta_{11}=0.084,\delta_{12}=0.084.$$

**Step 6.** Calculate the weighted evaluation value for the scoring for importance of indicators with Equation (16).

*η*_1_ = (0.69,0.23), *η*_2_ = (0.83,0.11), *η*_3_ = (0.73,0.21), *η*_4_ = (0.73,0.20), *η*_5_ = (0.80,0.14), *η*_6_ = (0.82,0.11), *η*_7_ = (0.87,0.07), *η*_8_ = (0.84,0.10), *η*_9_ = (0.77,0.15), *η*_10_ = (0.66,0.26), *η*_11_ = (0.75,0.18), *η*_12_ = (0.87,0.08), *η*_13_ = (0.85,0.09), *η*_14_ = (0.83,0.10), *η*_15_ = (0.83,0.10), *η*_16_ = (0.77,0.16), *η*_17_ = (0.78,0.16), *η*_18_ = (0.80,0.14), *η*_19_ = (0.82,0.12), *η*_20_ = (0.78,0.15), *η*_21_ = (0.81,0.13), *η*_22_ = (0.86,0.08), *η*_23_ = (0.79,0.14), *η*_24_ = (0.70,0.23), *η*_25_ = (0.64,0.29), *η*_26_ = (0.74,0.19), *η*_27_ = (0.53,0.36).

**Step 7.** Obtain the weight of indicators with Equation (17).

*β*_1_ = (0.04,0.95), *β*_2_ = (0.06,0.92), *β*_3_ = (0.05,0.94), *β*_4_ = (0.05,0.94), *β*_5_ = (0.06,0.93), *β*_6_ = (0.06,0.92), *β*_7_ = (0.07,0.91), *β*_8_ = (0.06,0.92), *β*_9_ = (0.05,0.93), *β*_10_ = (0.04,0.95), *β*_11_ = (0.05,0.94), *β*_12_ = (0.07,0.91), *β*_13_ = (0.07,0.92), *β*_14_ = (0.06,0.92), *β*_15_ = (0.07,0.92), *β*_16_ = (0.05,0.94), *β*_17_ = (0.06,0.93), *β*_18_ = (0.06,0.93), *β*_19_ = (0.06,0.92), *β*_20_ = (0.05,0.93), *β*_21_ = (0.06,0.93), *β*_22_ = (0.07,0.91), *β*_23_ = (0.06,0.93), *β*_24_ = (0.05,0.94), *β*_25_ = (0.04,0.95), *β*_26_ = (0.05,0.94), *β*_27_ = (0.03,0.96).

**Step 8.** Let k=1/2. Calculate the score value of the weight of indicators with Equation (19), as is shown in Table 6.

**Figure 1:**
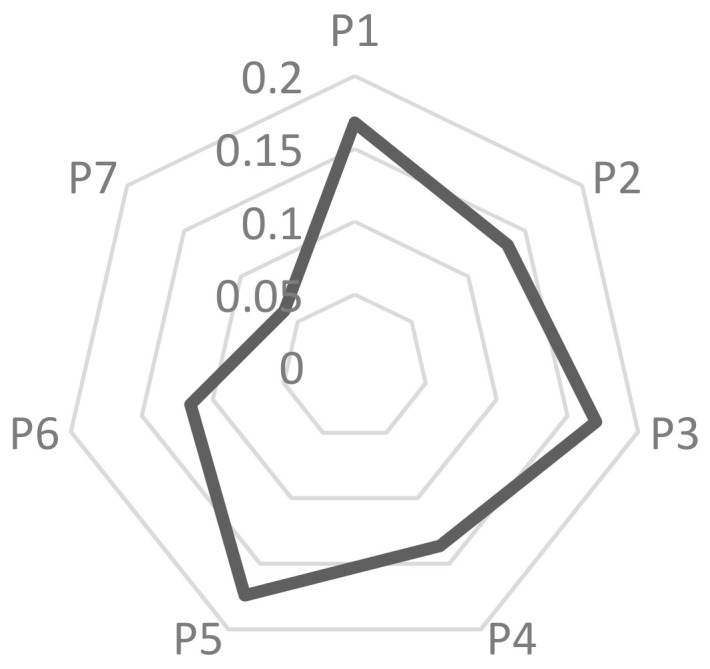
Evaluation results of seven enterprises.

**Steps 9 and 10.** Let k=1/2, obtain the final evaluation results with Equation (19) and calculate the final evaluation score of enterprises with Equation (20). The results are shown in Table 7 and Figure 1.

### 5.2. Discussion

According to Table 6, it can be seen that among the 27 secondary indicators, the most important indicator is the degree of disclosure of pollutant discharge compliance. This is mainly because the indicator system of this paper is used to evaluate the quality of sustainability information disclosure in heavy polluting industries. Stakeholders in heavily polluting industries are highly concerned about their pollutant emissions. The second important indicator is the authenticity of disclosure. There is information asymmetry between companies and stakeholders. Stakeholders mainly understand the company through the information disclosed by the company. Therefore, the authenticity of the company’s disclosure content is particularly important. In addition, the importance of indicators such as the substantial disclosure of major environmental problems and the timeliness of disclosure is also high. Relatively speaking, the innovative of disclosure and the systematic of disclosure are not very important. It can be seen that the attention paid to the language of the disclosure is not high.

According to Table 7 and Figure 1, it can be seen that among the seven case companies, the highest quality of sustainability information disclosure is Sinopec Group, followed by Baoshan Iron & Steel Co., Ltd., and Zijin Mining Group again. The disclosure quality of Shaanxi Coal and Chemical Industry Group, Shandong Hongqiao and Conch Cement Co. Ltd. is medium. The quality of Boway Alloy Material Co., Ltd.’s disclosure is the lowest, and its score is far from that of other companies, this shows that the quality of sustainability information disclosure between different enterprises is still wide. The low quality of Boway Alloy Material Co., Ltd. information disclosure is greatly affected by its disclosure method, which only uses a small amount of supplementary disclosure in its annual report. Except for Boway Alloy Material Co., Ltd., the other six companies have issued independent reports to disclose their sustainability information, which greatly enhances the quality of sustainability information disclosure. In addition, during the research, it was found that even companies with high quality of sustainability information disclosure like Sinopec Group disclosed only one page of disclosure on the rupture and explosion of oil pipelines in 2013. It can be seen that there is a problem of simplifying the disclosure of negative information at the time of disclosure. At the time of the study, it was found that the sustainability information of most enterprises was not audited by independent third parties, which shows that the reliability of the disclosure information is questionable. Moreover, the heavily polluting industries should focus on their environmental impact at the time of disclosure, but it was found that their environmental disclosure was not sufficient.

Based on the above analysis, suggestions for sustainability information disclosure in heavily polluting industries are proposed: (1) The state should formulate uniform disclosure standards to avoid the large difference in the quality of sustainability information disclosure between different companies; (2) When companies disclose sustainability information, they should use independent reports to disclose them; (3) The company should disclose the negative information in detail to reduce the degree of information asymmetry; (4) The company’s sustainability information should be audited by an independent third party; (5) Heavy polluting industries should increase the disclosure of their environmental information.

## 6. Conclusions

This paper proposed an approach to evaluating the quality of sustainability information disclosure of listed companies in heavy polluting industries. It is evaluated from the aspects of completeness, adequacy, relevance, reliability, normativeness and clarity. Considering the ambiguity and complexity of evaluation information, intuitionistic fuzzy sets is applied to model linguistic ratings and entropy is used to derive the weight of experts, the object weight and the subject weight of the indicators. The corresponding method is proposed to aggregate the evaluation information. The case study verifies the proposed approach. It also shows that most heavily polluting companies do a good job in sustainability information disclosure, but there is still a large gap in the quality of sustainability information disclosure between different companies. From this study, the quality of the disclosure of sustainability information can be evaluated more comprehensive. The six evaluation aspects along with the criteria can be valuable references for the research of disclosure of other information. The proposed evaluation method can effectively integrate experts’ opinions. Entropy plays a more important role in it. Through the entropy, the importance of expert opinions is distinguished, and the weights of indicators are measured. The study provides a new application field of entropy. The proposed evaluation method has good applicability and enriches the research on decision making. It is universal and can be used to aggregate the ratings in the field of linguistic evaluation.

With the proposed approach, the quality of disclosure of sustainability information can be grasped comprehensively and directly. It will be the value basis of future improvement for disclosing the sustainability information and have important influence on the stakeholders’ decision making. In the future research, the proposed approach can be used in more companies to make further verification and improvements.

## Figures and Tables

**Table 1:** Quality Evaluation Indicator System for Sustainability information Disclosure.

Aspects	Indicators
Completeness (B_1_) [16]	The length of the sustainability report (C_1_)
	Degree of disclosure of negative information (C_2_) [8]
	Degree of disclosure of economic information (C_3_) [12]
	Degree of disclosure of social information (C_4_) [12]
	Degree of disclosure of corporate environmental policies and objectives (C_5_) [55]
	Degree of disclosure of corporate environmental responsibility and obligations (C_6_) [55]
	Degree of disclosure of pollutant discharge compliance (C_7_) [16]
	The degree of disclosure of the completion of the total emission reduction task (C_8_) [16]
	The degree of disclosure of the implementation of the “three simultaneous” system (C_9_) [16]
Adequacy (B_2_) [8]	Environmental information space accounts for the proportion of sustainable development report (C_10_)
	Method of information disclosure (consolidated report, supplementary report or independent report) (C_11_) [16]
	Substantial disclosure of major environmental problems (C_12_) [8]
	Substantial disclosure of environmental risks (C_13_)
	Substantial disclosure of environmental impact (C_14_) [8]
Relevance (B_3_) [17]	Timeliness of disclosure (C_15_) [17]
	Expected environmental risk (C_16_)
	The importance of disclosure (C_17_) [52]
Reliability (B_4_) [17,53]	The degree of disclosure of the third-party audit (C_18_)
	The degree of disclosure of the ISO environmental system certification (C_19_) [55]
	Neutrality of disclosure (C_20_) [17]
	Verifiability of disclosure (C_21_) [17]
	Authenticity of disclosure (C_22_) [17]
Normativeness (B_5_) [8,18]	The degree of disclosure of the GRI indicator index (C_23_) [8]
	The normalization and rigor of language expression (C_24_)
	Systematic of disclosure (C_25_)
Clarity (B_6_) [17]	Readability of disclosure (C_26_) [17,54]
	Innovative of disclosure (C_27_) [17]

**Table 2:** Linguistic term sets.

Linguistic Terms for Weight of Indicators	Linguistic Terms for Quality Level	IFNs
Very important (VI)	Very high (VH)	(0.90, 0.05, 0.05)
Importance (I)	High (H)	(0.75, 0.20, 0.05)
Medium (F)	Medium (F)	(0.50, 0.40, 0.10)
Unimportance (U)	Low (L)	(0.25, 0.60,0.15)
Very unimportant (VU)	Very low (VL)	(0.10, 0.80, 0.10)

**Table 3:** The intuitionistic fuzzy decision matrix of weight of indicators.

	$c_{1}\;$	$\;c_{2}\;$	$\;c_{3}\;$	$\;c_{4}\;$	$\;c_{5}\;$	$\;c_{6}\;$	$\;c_{7}\;$	$\;c_{8}\;$	$\;c_{9}\;$
$e_{1}$	(0.5,0.4)	(0.75,0.2)	(0.5,0.4)	(0.75,0.2)	(0.9,0.05)	(0.75,0.2)	(0.9,0.05)	(0.9,0.05)	(0.9,0.05)
$e_{2}$	(0.75,0.2)	(0.25,0.6)	(0.5,0.4)	(0.5,0.4)	(0.75,0.2)	(0.75,0.2)	(0.75,0.2)	(0.75,0.2)	(0.5,0.4)
$e_{3}$	(0.75,0.2)	(0.9,0.05)	(0.75,0.2)	(0.5,0.4)	(0.75,0.2)	(0.9,0.05)	(0.9,0.05)	(0.75,0.2)	(0.9,0.05)
$e_{4}$	(0.75,0.2)	(0.9,0.05)	(0.75,0.2)	(0.5,0.4)	(0.5,0.4)	(0.5,0.4)	(0.75,0.2)	(0.75,0.2)	(0.5,0.4)
$e_{5}$	(0.25,0.6)	(0.75,0.2)	(0.75,0.2)	(0.75,0.2)	(0.5,0.4)	(0.75,0.2)	(0.9,0.05)	(0.9,0.05)	(0.5,0.4)
$e_{6}$	(0.75,0.2)	(0.9,0.05)	(0.5,0.4)	(0.9,0.05)	(0.9,0.05)	(0.9,0.05)	(0.9,0.05)	(0.9,0.05)	(0.9,0.05)
$e_{7}$	(0.9,0.05)	(0.9,0.05)	(0.75,0.2)	(0.75,0.2)	(0.75,0.2)	(0.5,0.4)	(0.9,0.05)	(0.75,0.2)	(0.75,0.2)
$e_{8}$	(0.5,0.4)	(0.9,0.05)	(0.9,0.05)	(0.9,0.05)	(0.9,0.05)	(0.9,0.05)	(0.9,0.05)	(0.9,0.05)	(0.9,0.05)
$e_{9}$	(0.5,0.4)	(0.9,0.05)	(0.75,0.2)	(0.75,0.2)	(0.75,0.2)	(0.9,0.05)	(0.9,0.05)	(0.9,0.05)	(0.9,0.05)
$e_{10}$	(0.5,0.4)	(0.75,0.2)	(0.75,0.2)	(0.75,0.2)	(0.9,0.05)	(0.9,0.05)	(0.9,0.05)	(0.75,0.2)	(0.5,0.4)
$e_{11}$	(0.9,0.05)	(0.75,0.2)	(0.75,0.2)	(0.5,0.4)	(0.75,0.2)	(0.9,0.05)	(0.75,0.2)	(0.75,0.2)	(0.5,0.4)
$e_{12}$	(0.5,0.4)	(0.75,0.2)	(0.75,0.2)	(0.75,0.2)	(0.75,0.2)	(0.75,0.2)	(0.9,0.05)	(0.9,0.05)	(0.75,0.2)
	$c_{10}$	$c_{11}$	$c_{12}$	$c_{13}$	$c_{14}$	$c_{15}$	$c_{16}$	$c_{17}$	$c_{18}$
$e_{1}$	(0.5,0.4)	(0.75,0.2)	(0.9,0.05)	(0.9,0.05)	(0.9,0.05)	(0.9,0.05)	(0.75,0.2)	(0.75,0.2)	(0.9,0.05)
$e_{2}$	(0.75,0.2)	(0.75,0.2)	(0.75,0.2)	(0.25,0.6)	(0.25,0.6)	(0.25,0.6)	(0.25,0.6)	(0.5,0.4)	(0.25,0.6)
$e_{3}$	(0.5,0.4)	(0.5,0.4)	(0.9,0.05)	(0.9,0.05)	(0.75,0.2)	(0.75,0.2)	(0.9,0.05)	(0.75,0.2)	(0.75,0.2)
$e_{4}$	(0.75,0.2)	(0.5,0.4)	(0.9,0.05)	(0.75,0.2)	(0.75,0.2)	(0.9,0.05)	(0.75,0.2)	(0.75,0.2)	(0.75,0.2)
$e_{5}$	(0.25,0.6)	(0.5,0.4)	(0.75,0.2)	(0.9,0.05)	(0.9,0.05)	(0.75,0.2)	(0.5,0.4)	(0.75,0.2)	(0.75,0.2)
$e_{6}$	(0.9,0.05)	(0.9,0.05)	(0.9,0.05)	(0.9,0.05)	(0.9,0.05)	(0.9,0.05)	(0.75,0.2)	(0.9,0.05)	(0.75,0.2)
$e_{7}$	(0.5,0.4)	(0.5,0.4)	(0.75,0.2)	(0.75,0.2)	(0.9,0.05)	(0.5,0.4)	(0.75,0.2)	(0.5,0.4)	(0.75,0.2)
$e_{8}$	(0.5,0.4)	(0.75,0.2)	(0.9,0.05)	(0.9,0.05)	(0.9,0.05)	(0.9,0.05)	(0.9,0.05)	(0.9,0.05)	(0.9,0.05)
$e_{9}$	(0.75,0.2)	(0.9,0.05)	(0.9,0.05)	(0.9,0.05)	(0.9,0.05)	(0.75,0.2)	(0.9,0.05)	(0.75,0.2)	(0.75,0.2)
$e_{10}$	(0.75,0.2)	(0.75,0.2)	(0.9,0.05)	(0.9,0.05)	(0.75,0.2)	(0.9,0.05)	(0.75,0.2)	(0.75,0.2)	(0.9,0.05)
$e_{11}$	(0.75,0.2)	(0.9,0.05)	(0.9,0.05)	(0.9,0.05)	(0.5,0.4)	(0.9,0.05)	(0.75,0.2)	(0.9,0.05)	(0.9,0.05)
$e_{12}$	(0.5,0.4)	(0.75,0.2)	(0.75,0.2)	(0.75,0.2)	(0.9,0.05)	(0.9,0.05)	(0.75,0.2)	(0.75,0.2)	(0.75,0.2)
	$c_{19}$	$c_{20}$	$c_{21}$	$c_{22}$	$c_{23}$	$c_{24}$	$c_{25}$	$c_{26}$	$c_{27}$
$e_{1}$	(0.9,0.05)	(0.5,0.4)	(0.75,0.2)	(0.75,0.2)	(0.5,0.4)	(0.75,0.2)	(0.5,0.4)	(0.5,0.4)	(0.25,0.6)
$e_{2}$	(0.5,0.4)	(0.5,0.4)	(0.5,0.4)	(0.5,0.4)	(0.5,0.4)	(0.5,0.4)	(0.5,0.4)	(0.25,0.6)	(0.5,0.4)
$e_{3}$	(0.75,0.2)	(0.9,0.05)	(0.75,0.2)	(0.9,0.05)	(0.5,0.4)	(0.5,0.4)	(0.25,0.6)	(0.75,0.2)	(0.25,0.6)
$e_{4}$	(0.9,0.05)	(0.75,0.2)	(0.9,0.05)	(0.9,0.05)	(0.9,0.05)	(0.75,0.2)	(0.75,0.2)	(0.75,0.2)	(0.5,0.4)
$e_{5}$	(0.75,0.2)	(0.5,0.4)	(0.75,0.2)	(0.75,0.2)	(0.5,0.4)	(0.25,0.6)	(0.5,0.4)	(0.5,0.4)	(0.25,0.6)
$e_{6}$	(0.75,0.2)	(0.75,0.2)	(0.9,0.05)	(0.9,0.05)	(0.75,0.2)	(0.75,0.2)	(0.75,0.2)	(0.75,0.2)	(0.5,0.4)
$e_{7}$	(0.75,0.2)	(0.75,0.2)	(0.9,0.05)	(0.9,0.05)	(0.9,0.05)	(0.75,0.2)	(0.75,0.2)	(0.9,0.05)	(0.5,0.4)
$e_{8}$	(0.9,0.05)	(0.9,0.05)	(0.75,0.2)	(0.9,0.05)	(0.9,0.05)	(0.9,0.05)	(0.5,0.4)	(0.9,0.05)	(0.25,0.6)
$e_{9}$	(0.75,0.2)	(0.9,0.05)	(0.75,0.2)	(0.9,0.05)	(0.75,0.2)	(0.75,0.2)	(0.75,0.2)	(0.75,0.2)	(0.75,0.2)
$e_{10}$	(0.75,0.2)	(0.9,0.05)	(0.9,0.05)	(0.9,0.05)	(0.9,0.05)	(0.75,0.2)	(0.75,0.2)	(0.75,0.2)	(0.5,0.4)
$e_{11}$	(0.9,0.05)	(0.75,0.2)	(0.75,0.2)	(0.9,0.05)	(0.75,0.2)	(0.5,0.4)	(0.5,0.4)	(0.75,0.2)	(0.9,0.05)
$e_{12}$	(0.9,0.05)	(0.75,0.2)	(0.75,0.2)	(0.75,0.2)	(0.9,0.05)	(0.75,0.2)	(0.75,0.2)	(0.75,0.2)	(0.5,0.4)

**Table 4:** The weight of experts on the enterprise score.

	$\;e_{1}\;$	$\;e_{2}\;$	$\;e_{3}\;$	$\;e_{4}\;$	$\;e_{5}\;$	$\;e_{6}\;$	$\;e_{7}\;$	$\;e_{8}\;$	$\;e_{9}\;$	$\;e_{10}\;$	$\;e_{11}\;$	$e_{12}\;$
$P_{1}$	0.081	0.080	0.088	0.081	0.083	0.081	0.082	0.086	0.086	0.081	0.082	0.088
$P_{2}$	0.083	0.080	0.085	0.082	0.080	0.081	0.082	0.088	0.085	0.081	0.086	0.087
$P_{3}$	0.080	0.082	0.087	0.079	0.081	0.085	0.081	0.089	0.085	0.086	0.081	0.084
$P_{4}$	0.083	0.081	0.087	0.080	0.080	0.081	0.082	0.088	0.085	0.080	0.084	0.088
$P_{5}$	0.084	0.082	0.085	0.081	0.078	0.086	0.081	0.086	0.086	0.081	0.084	0.086
$P_{6}$	0.083	0.082	0.086	0.081	0.081	0.084	0.082	0.084	0.086	0.080	0.085	0.087
$P_{7}$	0.083	0.080	0.084	0.082	0.081	0.087	0.080	0.085	0.084	0.085	0.083	0.086

**Table 5:** The weighted evaluation value for the scoring of enterprise.

	$\;c_{1}\;$	$\;c_{2}\;$	$\;c_{3}\;$	$\;c_{4}\;$	$\;c_{5}\;$	$\;c_{6}\;$	$\;c_{7}\;$	$\;c_{8}\;$	$\;c_{9}\;$
$P_{1}$	(0.74,0.19)	(0.40,0.50)	(0.63,0.29)	(0.77,0.18)	(0.64,0.28)	(0.72,0.20)	(0.68,0.24)	(0.62,0.29)	(0.71,0.22)
$P_{2}$	(0.63,0.28)	(0.27,0.60)	(0.49,0.41)	(0.64,0.29)	(0.66,0.27)	(0.64,0.29)	(0.66,0.25)	(0.41,0.47)	(0.33,0.55)
$P_{3}$	(0.71,0.20)	(0.27,0.61)	(0.59,0.31)	(0.72,0.21)	(0.75,0.17)	(0.68,0.24)	(0.66,0.25)	(0.59,0.30)	(0.39,0.49)
$P_{4}$	(0.57,0.33)	(0.39,0.52)	(0.60,0.31)	(0.71,0.22)	(0.64,0.29)	(0.65,0.27)	(0.53,0.36)	(0.49,0.41)	(0.37,0.51)
$P_{5}$	(0.74,0.20)	(0.45,0.44)	(0.59,0.33)	(0.72,0.21)	(0.72,0.22)	(0.67,0.27)	(0.62,0.31)	(0.60,0.32)	(0.48,0.42)
$P_{6}$	(0.55,0.36)	(0.18,0.69)	(0.55,0.36)	(0.73,0.20)	(0.55,0.36)	(0.61,0.31)	(0.58,0.32)	(0.55,0.36)	(0.35,0.53)
$P_{7}$	(0.18,0.69)	(0.29,0.59)	(0.66,0.24)	(0.52,0.37)	(0.43,0.46)	(0.47,0.42)	(0.34,0.55)	(0.34,0.55)	(0.38,0.51)
	$c_{10}$	$c_{11}$	$c_{12}$	$c_{13}$	$c_{14}$	$c_{15}$	$c_{16}$	$c_{17}$	$c_{18}$
$P_{1}$	(0.67,0.25)	(0.64,0.28)	(0.48,0.43)	(0.40,0.46)	(0.51,0.38)	(0.68,0.26)	(0.44,0.45)	(0.71,0.22)	(0.50,0.40)
$P_{2}$	(0.68,0.24)	(0.64,0.27)	(0.34,0.55)	(0.34,0.55)	(0.40,0.47)	(0.60,0.32)	(0.47,0.42)	(0.57,0.35)	(0.43,0.47)
$P_{3}$	(0.77,0.16)	(0.74,0.19)	(0.37,0.52)	(0.42,0.47)	(0.46,0.42)	(0.71,0.23)	(0.45,0.44)	(0.69,0.23)	(0.54,0.37)
$P_{4}$	(0.55,0.36)	(0.64,0.28)	(0.35,0.53)	(0.31,0.55)	(0.33,0.54)	(0.71,0.22)	(0.44,0.45)	(0.61,0.31)	(0.46,0.43)
$P_{5}$	(0.59,0.32)	(0.72,0.20)	(0.49,0.41)	(0.43,0.46)	(0.43,0.46)	(0.69,0.24)	(0.44,0.45)	(0.65,0.28)	(0.60,0.32)
$P_{6}$	(0.60,0.31)	(0.63,0.29)	(0.23,0.64)	(0.29,0.57)	(0.25,0.60)	(0.69,0.25)	(0.33,0.54)	(0.58,0.33)	(0.41,0.48)
$P_{7}$	(0.27,0.60)	(0.36,0.53)	(0.33,0.55)	(0.38,0.50)	(0.36,0.52)	(0.58,0.34)	(0.46,0.43)	(0.52,0.38)	(0.48,0.42)
	$c_{19}$	$c_{20}$	$c_{21}$	$c_{22}$	$c_{23}$	$c_{24}$	$c_{25}$	$c_{26}$	$c_{27}$
$P_{1}$	(0.67,0.25)	(0.52,0.38)	(0.64,0.28)	(0.63,0.30)	(0.45,0.45)	(0.69,0.25)	(0.71,0.22)	(0.77,0.18)	(0.53,0.38)
$P_{2}$	(0.73,0.19)	(0.57,0.35)	(0.62,0.31)	(0.66,0.26)	(0.57,0.33)	(0.65,0.28)	(0.66,0.28)	(0.72,0.20)	(0.64,0.28)
$P_{3}$	(0.74,0.19)	(0.64,0.27)	(0.62,0.31)	(0.65,0.28)	(0.66,0.26)	(0.76,0.19)	(0.71,0.22)	(0.76,0.19)	(0.74,0.19)
$P_{4}$	(0.70,0.22)	(0.58,0.34)	(0.58,0.34)	(0.58,0.33)	(0.60,0.31)	(0.65,0.27)	(0.62,0.31)	(0.76,0.18)	(0.64,0.27)
$P_{5}$	(0.76,0.18)	(0.67,0.24)	(0.67,0.27)	(0.68,0.24)	(0.67,0.27)	(0.71,0.23)	(0.69,0.25)	(0.76,0.18)	(0.70,0.22)
$P_{6}$	(0.63,0.28)	(0.55,0.37)	(0.64,0.27)	(0.61,0.31)	(0.53,0.37)	(0.69,0.25)	(0.64,0.29)	(0.69,0.25)	(0.49,0.41)
$P_{7}$	(0.28,0.60)	(0.56,0.34)	(0.52,0.38)	(0.,0.28)	(0.27,0.60)	(0.62,0.31)	(0.55,0.37)	(0.65,0.28)	(0.33,0.56)

**Table 6:** The score value of the weight of indicators.

$\;s\left(\beta_{j}\right)\;$	Score	Rank	$\;s\left(\beta_{j}\right)\;$	Score	Rank	$\;s\left(\beta_{j}\right)\;$	Score	Rank
$s\left(\beta_{1}\right)$	−0.452	24	$s\left(\beta_{10}\right)$	−0.456	25	$s\left(\beta_{19}\right)$.	−0.429	8
$s\left(\beta_{2}\right)$	−0.431	9	$s\left(\beta_{11}\right)$	−0.443	18	$s\left(\beta_{20}\right)$	−0.439	15
$s\left(\beta_{3}\right)$	−0.449	22	$s\left(\beta_{12}\right)$	−0.422	3	$s\left(\beta_{21}\right)$	−0.434	11
$s\left(\beta_{4}\right)$	−0.445	21	$s\left(\beta_{13}\right)$	−0.426	5	$s\left(\beta_{22}\right)$	−0.420	2
$s\left(\beta_{5}\right)$	−0.435	12	$s\left(\beta_{14}\right)$	−0.431	10	$s\left(\beta_{23}\right)$	−0.438	14
$s\left(\beta_{6}\right)$	−0.429	7	$s\left(\beta_{15}\right)$	−0.425	4	$s\left(\beta_{24}\right)$	−0.450	23
$s\left(\beta_{7}\right)$	−0.416	1	$s\left(\beta_{16}\right)$	−0.443	20	$s\left(\beta_{25}\right)$	−0.458	26
$s\left(\beta_{8}\right)$	−0.427	6	$s\left(\beta_{17}\right)$	−0.439	16	$s\left(\beta_{26}\right)$	−0.443	19
$s\left(\beta_{9}\right)$	−0.441	17	$s\left(\beta_{18}\right)$	−0.437	13	$s\left(\beta_{27}\right)$	−0.468	27

**Table 7:** Evaluation results of seven enterprises.

	$\;P_{1}\;$	$\;P_{2}\;$	$\;P_{3}\;$	$\;P_{4}\;$	$\;P_{5}\;$	$\;P_{6}\;$	$\;P_{7}\;$
Evaluation results	(0.60,0.27)	(0.57,0.30)	(0.61,0.26)	(0.57,0.29)	(0.61,0.26)	(0.54,0.31)	(0.48,0.36)
Score value	0.168036	0.134568	0.170304	0.136327	0.17417	0.115692	0.061866
Rank	3	5	2	4	1	6	7

## References

[1] Ramírez-Carrillo E., López-Corona O., Toledo-Roy J.C., Lovett J.C., de León-González F., Osorio-Olvera L., Pérez-Cirera V. (2018). Assessing sustainability in North America’s ecosystems using criticality and information theory. bioRxiv.

[2] Van Schalkwyk R.F., Reuter M.A., Gutzmer J., Stelter M. (2018). Challenges of digitalizing the circular economy: Assessment of the state-of-the-art of metallurgical carrier metal platform for lead and its associated technology elements. J. Clean. Prod..

[3] Liu X., Liu H., Chen J., Liu T., Deng Z. (2018). Evaluating the sustainability of marine industrial parks based on the DPSIR framework. J. Clean. Prod..

[4] Amran A., Ooi S.K. (2014). Sustainability reporting: Meeting stakeholder demands. Eur. J. Mark..

[5] Nobanee H., Ellili N. (2016). Corporate sustainability disclosure in annual reports: Evidence from UAE banks: Islamic versus conventional. Renew. Sust. Energy Rev..

[6] Brammer S., Pavelin S. (2008). Factors influencing the quality of corporate environmental disclosure. Bus. Strategy Environ..

[7] Orazalin N., Mahmood M. (2018). Economic, environmental, and social performance indicators of sustainability reporting: Evidence from the Russian oil and gas industry. Energy Policy.

[8] Xu Y., Zhang C. (2015). Case Analysis of Environmental Performance Information Disclosure of Heavy Pollution Industry Based on Sustainability Report. Financ. Account. Mon..

[9] Martínez-Ferrero J., Garcia-Sanchez I.M., Cuadrado-Ballesteros B. (2015). Effect of financial reporting quality on sustainability information disclosure. Corpor. Soc. Respons. Environ. Manag..

[10] Michelon G., Parbonetti A. (2012). The effect of corporate governance on sustainability disclosure. J. Manag. Gover..

[11] Cuadrado-Ballesteros B., Frías-Aceituno J., Martínez-Ferrero J. (2014). The role of media pressure on the disclosure of sustainability information by local governments. Online Inf. Rev..

[12] Zhang C., Xu Y., Liu M. (2016). Evaluation of Sustainability information Disclosure of Listed Companies in Heavy Pollution Industry Based on Non-financial Reports. J. Nanjing Univ. Technol..

[13] Romolini A., Gori E., Fissi S. (2015). Quality disclosure in sustainability reporting: Evidence from universities. Transylv. Rev. Admin. Sci..

[14] Manes-Rossi F., Tiron-Tudor A., Nicolò G., Zanellato G. (2018). Ensuring More Sustainable Reporting in Europe Using Non-Financial Disclosure—De Facto and De Jure Evidence. Sustainability.

[15] Dilling P.F. (2010). Sustainability Reporting in A Global Context: What Are the Characteristics of Corporations That Provide High Quality Sustainability Reports—An Empirical Analysis. Int. J. Econ.. Bus. Res..

[16] Feng H., Wang Y. (2012). Research on Environmental Information Disclosure of Listed Companies in China’s Heavy Pollution Industry. Product. Res..

[17] Yin K., Liu X., Li H. (2013). Construction of Social Responsibility Information Disclosure Quality Evaluation and Construction of Quality Standards. Friends Account..

[18] Daub C.H. (2007). Assessing the Quality of Sustainability Reporting: An Alternative Methodological Approach. J. Clean. Prod..

[19] Garg H., Kaur J. (2018). A Novel (*R,S*)-Norm Entropy Measure of Intuitionistic Fuzzy Sets and Its Applications in Multi-Attribute Decision-Making. Mathematics.

[20] Kong D., Chang T., Wang Q., Sun H., Dai W. (2018). A threat assessment method of group targets based on interval-valued intuitionistic fuzzy multi-attribute group decision-making. Appl. Soft Comput..

[21] Bao T., Xie X., Long P., Wei Z. (2017). MADM method based on prospect theory and evidential reasoning approach with unknown attribute weights under intuitionistic fuzzy environment. Expert Syst. Appl..

[22] Cavallaro F., Zavadskas E.K., Streimikiene D. (2018). Concentrated solar power (CSP) hybridized systems. Ranking based on an intuitionistic fuzzy multi-indicators algorithm. J. Clean. Prod..

[23] Li M., Wei W., Wang J., Qi X. (2018). Approach to Evaluating Accounting Informatization Based on Entropy in Intuitionistic Fuzzy Environment. Entropy.

[24] Zavadskas E.K., Podvezko V. (2016). Integrated determination of objective indicators weights in MCDM. Int. J. Inform. Tech. Decis. Mak..

[25] Zoraghi N., Amiri M., Talebi G., Zowghi M. (2013). A fuzzy MCDM model with objective and subjective weights for evaluating service quality in hotel industries. J. Indus. Eng. Int..

[26] Zhang S.F., Liu S.Y. (2011). A GRA-based intuitionistic fuzzy multi-indicators group decision making method for personnel selection. Expert Syst. Appl..

[27] Chen T.Y., Li C.H. (2011). Objective weights with intuitionistic fuzzy entropy measures and computational experiment analysis. Appl. Soft Comput. J..

[28] Zhang Y., Ma P., Su X., Zhang C. (2012). Interval Intuitionistic Fuzzy Multi-attribute Decision Making with Attribute Weight Uncertainty. J. Auto.

[29] Shen F., Xu J., Xu Z. (2016). An outranking sorting method for multi-indicators group decision making using intuitionistic fuzzy sets. Inf. Sci..

[30] Ouyang Y., Pedrycz W. (2016). A new model for intuitionistic fuzzy multi-attributes decision making. Eur. J. Oper. Res..

[31] Nguyen H. (2016). A new interval-valued knowledge measure for interval-valued intuitionistic fuzzy sets and application in decision making. Expert Syst. Appl..

[32] Liu P. (2017). Multiple attribute group decision making method based on interval-valued intuitionistic fuzzy power Heronian aggregation operators. Comput. Ind. Eng..

[33] Atanassov K.T. (1986). Intuitionistic fuzzy sets. Fuzzy Sets Syst..

[34] Liu M., Ren H. (2015). Research on Multi-attribute Decision Making Method Based on a New Class of Intuitionistic Fuzzy Entropy. Syst. Eng. Theory Pract..

[35] Zedeh L.A. (1965). Fuzzy set. Inform. Control.

[36] Rostamzadeh R., Esmaeili A., Nia A.S., Saparauskas J., Ghorabaee M.K. (2017). A Fuzzy Aras Method for Supply Chain Management Performance Measurement in SMEs under Uncertainty. Transform. Bus. Econ..

[37] Zhou J., Su W., Baležentis T., Streimikiene D. (2018). Multiple Indicators Group Decision-Making Considering Symmetry with Regards to the Positive and Negative Ideal Solutions via the Pythagorean Normal Cloud Model for Application to Economic Decisions. Symmetry.

[38] Li D.F. (2010). Linear programming method for MADM with interval-valued intuitionistic fuzzy sets. Expert Syst. Appl..

[39] Tian H., Li J., Zhang F., Xu Y., Cui C., Deng Y., Xiao S. (2018). Entropy Analysis on Intuitionistic Fuzzy Sets and Interval-Valued Intuitionistic Fuzzy Sets and Its Applications in Mode Assessment on Open Communities. J. Adv. Comput. Intell. Intell. Inform..

[40] Wang C., Yao D., Mao J., Sun L. (2012). Intuitionistic fuzzy multiple attribute decision making method based on entropy and correlation coefficient. Comput. Appl..

[41] Wang B., Liang K. (2013). Entropy-based supply chain partner risk assessment and selection method. Tech. Econ. Manag. Res..

[42] Cavallaro F., Zavadskas E.K., Raslanas S. (2016). Evaluation of combined heat and power (CHP) systems using fuzzy shannon entropy and fuzzy TOPSIS. Sustainability.

[43] Krylovas A., Dadelo S., Kosareva N., Zavadskas E.K. (2017). Entropy–KEMIRA Approach for MCDM Problem Solution in Human Resources Selection Task. Int. J. Inf. Technol. Decis. Making.

[44] Wu S., Fu Y., Shen H., Liu F. (2018). Using ranked weights and Shannon entropy to modify regional sustainable society index. Sustain. Cities Soc..

[45] Ramirezcarrillo E., Lopezcorona O., Toledoroy J.C., Lovett J., Leongonzalez F.D. (2018). Assessing sustainability in North America’s ecosystems using criticality and information theory. PLoS ONE.

[46] Liu H.W., Wang G.J. (2007). Multi-indicators decision-making methods based on intuitionistic fuzzy sets. Eur. J. Oper. Res..

[47] Nguyen H. (2015). A new knowledge-based measure for intuitionistic fuzzy sets and its application in multiple attribute group decision making. Expert Syst. Appl..

[48] Joshi D., Kumar S. (2016). Interval-valued intuitionistic hesitant fuzzy Choquet integral based TOPSIS method for multi-indicators group decision making. Eur. J. Oper. Res..

[49] Atanassov K.T. (1999). Intuitionistic fuzzy sets. Intuitionistic Fuzzy Sets.

[50] Xu Z. (2007). Intuitionistic fuzzy aggregation operators. IEEE Trans. Fuzzy syst..

[51] http://www.china.com.cn/policy/txt/2010-09/15/content_20933130_2.htm.

[52] Unerman J., Zappettini F. (2014). Incorporating Materiality Considerations into Analyses of Absence from Sustainability Reporting. Soc. Environ. Account. J..

[53] Li Y., Li N. (2014). Study on External Pressure, Firm Characteristics and the Quality of Environmental Information Disclosure of Listed Companies in China. Adv. Mater. Res..

[54] Qiu X., Jiang S., Deng K. (2013). Automatic Assessment of Information Disclosure Quality in Chinese Annual Reports. Commun. Comput. Inform. Sci..

[55] Yao C., Yu H. (2017). Analysis on the Status Quo of Environmental Information Disclosure of Listed Companies in Heavy Pollution Industry. Commer. Account..

[56] Wang X., Wei C., Guo T. (2011). Determination of Expert Weights for Intuitionistic Fuzzy Multi-Attribute Group Decision Making Based on Cross Entropy and Entropy. J. Qufu Normal Univ. (Nat. Sci. Ed.).

